# Tauroursodeoxycholic acid reduces glial cell activation in an animal model of acute neuroinflammation

**DOI:** 10.1186/1742-2094-11-50

**Published:** 2014-03-19

**Authors:** Natalia Yanguas-Casás, M Asunción Barreda-Manso, Manuel Nieto-Sampedro, Lorenzo Romero-Ramírez

**Affiliations:** 1 Laboratorio de Plasticidad Neural, Instituto Cajal (CSIC), Avenida Doctor Arce 37, 28002 Madrid, Spain; 2 Laboratorio de Plasticidad Neural, Unidad de Neurología Experimental, Hospital Nacional de Parapléjicos (SESCAM), Finca la Peraleda s/n, 45071 Toledo, Spain

**Keywords:** Astrocytes, Bile salts, Inducible nitric oxide synthase, Lipopolysaccharide, Microglia, Migration, Monocyte chemotactic protein-1, NFκB, Protein kinase RNA-activated, Vascular cell adhesion molecule 1

## Abstract

**Background:**

Bile acids are steroid acids found predominantly in the bile of mammals. The bile acid conjugate tauroursodeoxycholic acid (TUDCA) is a neuroprotective agent in different animal models of stroke and neurological diseases. However, the anti-inflammatory properties of TUDCA in the central nervous system (CNS) remain unknown.

**Methods:**

The acute neuroinflammation model of intracerebroventricular (icv) injection with bacterial lipopolysaccharide (LPS) in C57BL/6 adult mice was used herein. Immunoreactivity against Iba-1, GFAP, and VCAM-1 was measured in coronal sections in the mice hippocampus. Primary cultures of microglial cells and astrocytes were obtained from neonatal Wistar rats. Glial cells were treated with proinflammatory stimuli to determine the effect of TUDCA on nitrite production and activation of inducible enzyme nitric oxide synthase (iNOS) and NFκB luciferase reporters. We studied the effect of TUDCA on transcriptional induction of iNOS and monocyte chemotactic protein-1 (MCP-1) mRNA as well as induction of protein expression and phosphorylation of different proteins from the NFκB pathway.

**Results:**

TUDCA specifically reduces microglial reactivity in the hippocampus of mice treated by icv injection of LPS. TUDCA treatment reduced the production of nitrites by microglial cells and astrocytes induced by proinflammatory stimuli that led to transcriptional and translational diminution of the iNOS. This effect might be due to inhibition of the NFκB pathway, activated by proinflammatory stimuli. TUDCA decreased *in vitro* microglial migration induced by both IFN-γ and astrocytes treated with LPS plus IFN-γ. TUDCA inhibition of MCP-1 expression induced by proinflammatory stimuli could be in part responsible for this effect. VCAM-1 inmunoreactivity in the hippocampus of animals treated by icv LPS was reduced by TUDCA treatment, compared to animals treated with LPS alone.

**Conclusions:**

We show a triple anti-inflammatory effect of TUDCA on glial cells: i) reduced glial cell activation, ii) reduced microglial cell migratory capacity, and iii) reduced expression of chemoattractants (e.g., MCP-1) and vascular adhesion proteins (e.g., VCAM-1) required for microglial migration and blood monocyte invasion to the CNS inflammation site. Our results present a novel TUDCA anti-inflammatory mechanism, with therapeutic implications for inflammatory CNS diseases.

## Background

Central nervous system (CNS) homeostasis is maintained by the blood brain barrier (BBB) restricting the passage of substances and cells from the blood to the CNS parenchyma, as well as the active role of CNS resident cells (particularly astroglial and microglial cells), sensing and responding to any imbalance in the CNS environment. Infections, trauma, stroke, toxins, and other perturbations are capable of arousing an immediate short-term innate immune response as a defence mechanism to protect the CNS from insults. The response is resolved once the threat has been eliminated and homeostasis is restored. This acute neuroinflammatory response includes the activation of astrocytes [1] and the resident immune cells (microglia) [2]. When glial cells are activated, they change their morphology to the “reactive state”, increasing the expression of specific proteins (e.g., glial fibrillary acidic protein (GFAP) in astrocytes and ionized calcium-binding adapter molecule 1 (Iba-1) in microglia) and their migratory capacity to the insult site. Activated microglial cells increase the phagocytic activity. CNS glial cells can regulate this inflammatory response [3-5]. If the glial cells cannot restore the homeostasis, the inflammatory response is maintained long after the initial insult. This chronic neuroinflammation causes the loss of white and grey matter that leads to functional deficits [6,7] that characterize the pathology of neurodegenerative diseases [8,9], stroke [10], and traumatic brain injuries [11]. Reactive glial cells release a wide number of mediators, including proinflammatory and anti-inflammatory cytokines, and chemokines that increase BBB permeability and induce the activation and recruitment of blood monocytes, lymphocytes, and neutrophils to the inflammation site inside the CNS parenchyma [12,13].

Bile acids, such as ursodeoxycholic (UDCA) and its conjugated derivative tauroursodeoxycholic acid (TUDCA), have neuroprotective effects in several neurodegenerative diseases in neuronal culture [14] and in ischemia/reperfusion animal models, reducing infarct area and inflammation [15-18]. The anti-inflammatory effect of bile acids has been previously described in BV-2 microglial cells, reducing nitrite production after β-amyloid peptide treatment [19]. Bile acids are an interesting therapeutic tool since they can be administered either orally, intravenously, or intraperitoneally, and they easily cross the BBB. UDCA is an FDA approved drug for the treatment of primary biliary cirrhosis and has not shown any relevant side effects during chronic treatments [20].

In this study, we tested the *in vitro* anti-inflammatory effect of the bile salt TUDCA in the glial cells involved in neuroinflammation and in an animal model of acute brain inflammation.

## Methods

### Reagents

Tauroursodeoxycholic acid, sodium salt (TUDCA) was purchased from Calbiochem (La Jolla, CA, USA). *E. coli* lipopolysaccharides (LPS) isotypes 026:B6 and 055:B5, Roswell Park Memorial Institute medium 1640 (RPMI), Dulbecco’s modified Eagle’s medium (DMEM), penicillin/streptomycin mix (P/S), and poly-L-lysine were purchased from Sigma-Aldrich (St Louis, MO, USA). Foetal bovine serum (FBS) and horse serum were purchased from Gibco BRL (Gaithersburg, MD, USA).

### Acute brain inflammation in a mouse model

We used 8–10-week-old C57/BL6 mice purchased from Harlan® Interfauna Iberica (Sant-Feliu-de-Codines, Spain) to study acute brain inflammation. The animals were given food and water *ad libitum*, and were housed in the Cajal Institute animal house at a controlled ambient temperature of 22°C with 50% ± 10% relative humidity and with a 12 h light/dark cycle. Experiments were carried out in accordance with the Guidelines of the European Union Council (86/609/EU) and following the Spanish regulations (BOE 67/8509-12, 1988) for the use of laboratory animals, and were approved by the Ethics and Scientific Committees of Instituto Cajal, CSIC, and Hospital Nacional de Parapléjicos, SESCAM.

Two experimental procedures were used to determine the effect of TUDCA on acute brain inflammation: in the first procedure, 21 mice were anesthetized with 3 mL/kg of equitesin and 2 mg/kg LPS from *E. coli* isotype 055:B5 (Sigma-Aldrich, St Louis, MO, USA), diluted in 5 μL of phosphate-buffered saline (PBS), was injected intracerebroventricularly (icv) on the stereotaxic coordinates AP: -0.46, ML: -1.0, and DV: -1.8 from bregma [21] with a Hamilton syringe. One group of mice (n = 11) was treated with one intraperitoneal (ip) injection of TUDCA at 500 mg/kg every 8 h, starting right after the icv LPS injection. A control group of mice (n = 6) received an icv injection with 5 μL of PBS at the same coordinates. An additional group of untreated mice (n = 3) was used as a control to assess the inflammatory effect of the icv injections with PBS. Three days after the icv injection the animals were sacrificed with an overdose of sodium pentobarbital (50 mg/kg, ip), and perfused with 60 mL of saline buffer and 60 mL of 4% paraformaldehyde (PFA, MERCK, Darmstatd, Germany). Brains were extracted, post-fixed for 24 h in 4% PFA at 4°C, left for 48 h in 30% sucrose at 4°C, embedded in OCT™ Compound (Tissue-Tek®, Sakura Finetek Europe, Alphen aan den Rijn, The Netherlands) and stored at –20°C until further use.

In the second experimental procedure, we performed the same acute brain inflammation model on 26 mice, half of which (n = 13) received an icv injection with 5 μL of PBS and half of which (n = 13) received an icv injection with 5 μL of LPS. Seven mice from each experimental group were injected with TUDCA (500 mg/kg, ip) right after the icv injection at 3, 6, 9, and 23 h. Mice were sacrificed 24 h after the icv injection by cervical dislocation and brains were extracted, fixed in 4% PFA at 4°C for 48 h, then left for 72 h in 30% sucrose at 4°C and embedded in OCT™ compound, as described above. An additional group of untreated mice (n = 3) was processed as a control group.

### Immunohistochemistry

Serial sections (15-μm thick) from the hippocampus were cut on a cryostat LEICA CM1900 (Nussloch, Germany), mounted on gelatin-coated slides (n = 7 sections per slide) and stored at –20°C until further use. For immunolabeling, endogenous peroxidase activity was previously quenched with a solution of peroxide. After blocking with normal serum, sections were incubated overnight at 4°C with the primary antibody. A specific antibody against GFAP was used to detect astrocytes, anti-Iba-1 antibody was used to detect microglia, and an antibody against vascular cell adhesion molecule 1 (VCAM-1, for more details see Table 1 and Additional file 1) was used to stain endothelial cells. Slides were incubated for 90 min at room temperature with the corresponding biotinylated secondary antibody. The signal was amplified with Vectastain ABC reagent (Vectastain ABC kit, Vector Laboratories, Burlingame, CA, USA) and the immunohistochemical stain was developed with 3,3′-diaminobenzidine. Slides were mounted with DePeX mounting medium (BDH, Poole, England) and photographed using an Olympus Provis AX70 microscope, coupled to an Olympus PD50 photography system. Image J software (Wayne Rasband, NIH, USA) was used to obtain the photographs and analyse the images.

**Table 1:** Antibodies for immunohistochemistry

**Antibody**	**Host**	**Distributor**	**Working dilution**
Iba-1	Rabbit	WAKO	1:2000
GFAP (096)	Rabbit	DAKO	1:2000
VCAM-1 (P3C4)	Mouse	Iowa Hybridoma Bank	1:500
α-rabbit biotinylated	Goat	Jackson ImmunoResearch	1:200
α-mouse biotinylated	Goat	Jackson ImmunoResearch	1:200

### Cell culture

Primary cultures of microglial cells were obtained from newborn (P0) to 2-day-old (P2) Wistar rat forebrains and grown in DMEM medium supplemented with 10% heat-inactivated FBS, 10% heat-inactivated horse serum, and P/S (DMEM 10:10:1) in 75-cm^2^ flasks, coated with poly-L-lysine (10 μg/mL) [22]. Briefly, after reaching confluence, cells were shaken at 230 rpm for 3 h at 37°C. Detached cells were centrifuged at 168× *g* for 10 min. Cell pellets were resuspended in warm DMEM 10:10:1 and plated at a density of 200,000 cells/cm^2^. For experiments, microglial cells were resuspended in RPMI 1640 medium supplemented with 10% FBS and P/S.

Primary cultures of astrocytes were obtained from newborn (P0) to 2-day-old (P2) Wistar rat cortices [23]. The tissue homogenate was filtered through a 40-μm mesh (BD Falcon, Franklin Lakes, NJ, USA) and centrifuged at 950 rpm for 5 to 7 min. The pellet was plated and grown in DMEM supplemented with 10% FBS and P/S in 75-cm^2^ flasks coated with poly-L-lysine (10 μg/mL). Media was changed every 3 to 4 days. After reaching confluence, cultures were shaken overnight at 280 rpm and 37°C in a shaker (Infors Minitron Botmingen, Switzerland). Detached cells were washed off with PBS and the remaining astrocyte monolayer was trypsinized and replated at a density of 30,000 cells/cm^2^.

### Nitrite production assays

Inducible nitric oxide synthase (iNOS) activity in cell cultures was assessed by measuring nitrite accumulation in the cell culture media [24]. The optimal concentrations of LPS and IFN-γ used are shown in Additional file 2. We tested different *E. coli* LPS isotypes (055:B5 and 026:B6) at different concentrations and the effect of the presence/absence of IFN-γ in the treatment, since we did not find a consensus for microglia cells and astrocyte in the literature. Our results show that only the 026:B6 LPS isotype stimulated a proinflammatory response in rat microglial cells *in vitro*, whereas the 055:B5 isotype did not. The addition of IFN-γ did not increase this response. Therefore, we decided to use the 026:B6 isotype without IFN-γ for microglial treatment. However, astrocytes were stimulated with both LPS isotypes, but nitrite production was obtained only when we added LPS together with IFN-γ. To be consistent with both cell types we decided to perform the *in vitro* experiments with the 026:B6 isotype, at the optimal concentration for nitric oxide production for each cell type.

Cells were pretreated with different concentrations of TUDCA for 90 to 120 min and were then treated with LPS from *E. coli* isotype 026:B6 (200 ng/mL for microglial cells) or LPS plus IFN-γ (1 μg/mL LPS plus 20 ng/mL IFN-γ, for astrocytes) for an additional 24 h in low serum media (2% FBS). Supernatants were mixed with modified Griess reagent (Sigma-Aldrich, Saint Louis, MO, USA) (v/v, 1:1), shaken, and absorbance was measured at λ_492_ in a Multiskan Ascent (Thermo Electron Co., Shanghai, China).

Nitrite production was related to viable cells measured with 3-(4,5-dimethylthiazol-2-yl)-2,5-diphenyltetrazolium bromide (MTT) assay. After removing the conditioned media for nitrite determination, MTT (Sigma-Aldrich, Saint Louis, MO, USA) dissolved in DMEM or RPMI medium without phenol-red was added to the treated cells (0.5 μg/mL). After 3 h incubation at 37°C, cell culture media was removed and 100 μL of dimethyl sulfoxide (Sigma-Aldrich, Saint Louis, MO, USA) was added to each well, shaken, and the absorbance was measured at λ_595_ in the same device. Experiments were performed in triplicate and the assay repeated at least six times with microglial cells and at least four times with astrocytes.

### RNA purification and qPCR

Cell were pretreated with TUDCA (200 μM) for 2 h and were then treated with proinflammatory stimuli (200 ng/mL of LPS for microglial cells; 1 μg/mL LPS plus 10 ng/mL IFN-γ, for astrocytes) for 6 and 24 h. Gene and protein expression of untreated cells and untreated cells exposed to TUDCA were also determined. Total RNA for quantitative real-time PCR (qPCR) was isolated from cultured primary microglia cells and astrocytes with TRIzol reagent (Invitrogen, Carlsbad, CA, USA), extracted, and reverse transcribed with RevertAid™ H Minus First Strand cDNA Synthesis Kit (Fermentas, Vilnius, Lithuania). Specific primers for different RNA messengers (mRNA) were obtained with Primer Express 3.0 software (Applied Biosystems, Warrington, UK) and the pair of primers with less secondary structures for all the mRNA were selected (for more information see Table 2), once analyzed by Gene Runner 3.05 software (Hastings Software Inc.). Quantitative PCR was developed in a 7500 Real Time PCR System (Applied Biosystems, Warrington, UK) with Power SYBR® Green (Applied Biosystems) reagent. Gene expression was determined with 7500 Software v2.0.4 and the passive reference gene was ROX. Results are presented as the ratio between transcriptional expression of the gene of interest and the transcriptional expression of a housekeeping gene as a loading control. We tested the transcriptional expression of several housekeeping genes (18S ribosomal RNA, 36B4 ribosomal protein, and β-actin). Although we did not see any major differences among them, we decided to use β-actin as a normalized control for astrocytes and 36B4 for microglial cells.

**Table 2:** Primers for quantitative PCR

**Gene**	**Accession #**	**Forward primer 5′-3′**	**Reverse primer 5′-3′**	**Product length**
iNOS	NM_012611.3	acattgatctccgtgacagcc	cccttcaatggttggtacatg	158
MCP-1	NM_031530.1	tgctgtctcagccagatgcagtta	tacagcttctttgggacacctgct	131
β-actin	NM_031144.3	tccgtaaagacctctatgc	atcttcatggtgctaggagc	114
36B4	NM_022402.2	ttcccactggctgaaaaggt	cgcagccgcaaatgc	59

### Transient transfection experiments in glial cells with luciferase reporters

Microglial cells (300,000 cells/well) were seeded on 24-well plates coated with poly-L-lysine (50 μg/mL). After 24 h, cells were transfected using a transfection mixture, according to the manufacturer’s protocol, with a firefly luciferase reporter plasmid (1 μg/well), pSV40-Renilla luciferase plasmid (100 ng/well, Promega, Madison, WI, USA) as a control for transfection efficiency, and XtremeGENE HP DNA Transfection Reagent (1 μL/well, Roche, Indianapolis, IN, USA) in OPTIMEM. A rat iNOS-pGL3 firefly luciferase reporter plasmid containing a 720 bp fragment from the 5′ flanking region of the rat iNOS promoter [25] and a NFκB-pGL3 firefly reporter plasmid [26] containing a -241 to -54 base pair fragments of 5′ flanking region with the NFκB binding site from the human E-selectin promoter (Addgene plasmid #13029) were used. After 24 h of incubation, the transfection mixture was removed from the wells and cells were cultured overnight in culture media with low serum and treated with LPS (200 ng/mL) or TUDCA plus LPS for 6 h (for NFκB-pGL3 reporter) and 24 h (for iNOS-pGL3 reporter). After treatment, the media was removed from the wells and 100 μL/well of 1× Passive Lysis Buffer (Promega, Madison, WI, USA) was added. Culture plates were sealed with parafilm and stored at –80°C until luciferase activity determination.

Astrocytes (20,000 cells/well) were seeded on 96-well plates coated with poly-L-lysine (10 μg/mL). Cells were transfected by adding a firefly luciferase reporter plasmid (0.2 μg/well), a pSV40-Renilla luciferase plasmid (50 ng/well) as control for transfection efficiency, and XtremeGENE 9 DNA Transfection Reagent (0.4 μL/well, Roche) in OPTIMEM according to the manufacturer’s protocol. After 24 h of incubation, the transfection mixture was removed from the wells and the cells were cultured overnight in culture media with low serum and treated with LPS (1 μg/mL) and IFN-γ (10 ng/mL) or different concentrations of TUDCA with LPS plus and IFN-γ for 6 h (for ELAM-pGL3 reporter) and 24 h (for iNOS-pGL3 reporter). After treatment, the media was removed from the wells and 50 μL/well of 1× Passive Lysis Buffer was added. Culture plates were sealed with parafilm and stored at –80°C until luciferase determination. Laboratory-made dual-luciferase buffers were used. Firefly luciferase buffer (50 μL/sample of 30 mM Tricine, 0.1 mM EDTA pH 8, 15 mM magnesium sulfate, 10 mM DTT, 533.3 μM ATP, 0.4 mM D-Luciferin, and 0.27 mM Coenzyme A adjusted to pH 7.8) was mixed in a tube with the sample and luciferase activity was measured in a luminometer Sirius (Berthold). Renilla luciferase buffer (100 μL/sample of 0.22 M potassium phosphate pH 5.1, 1.1 M sodium chloride, 2.2 mM EDTA, 0.44 mg/mL BSA, 1.3 mM sodium azide, and 1.43 μM coelenterazine, adjusted to pH 5.0) was added to the same tube with the mix and the Renilla luciferase activity was measured again in the luminometer. Results are presented as the mean ± standard deviation (SD) of the fold induction related to the control of the ratio firefly luciferase activity/Renilla luciferase activity of at least three individual experiments in triplicate.

### Western blotting

Cells were washed with ice cold PBS and lysed in a buffer containing 50 mM Tris-HCl (pH 7.6), 137 mM NaCl, 0.5 mM DTT, 1% Nonidet-P40, 0.2% sodium dodecyl sulphate (SDS), 0.5 μM Okadaic acid, and Phosphatase and Protease Inhibitor Cocktail Tablets (PhosSTOP and cOmplete Mini, Roche). Protein samples (100 μg for microglial lysates and 50 μg for astroglial lysates) were dissolved into 10% SDS-polyacrylamide gel electrophoresis (SDS-PAGE) and wet-transferred overnight at 4°C to a nitrocellulose membrane (Whatman, GmbH, Dassel, Germany). Membranes were blocked with 5% (w/v) dry skimmed milk or BSA in TBS with 0.1% Tween 20 (TTBS) for 1 h at room temperature and incubated overnight at 4°C with the corresponding primary antibody (for more information, see Table 3). After washing with TTBS and TBS, membranes were incubated with HRP-conjugated secondary antibodies for 1 h at room temperature and the protein bands were detected using Supersignal west pico or west femto chemiluminescent substrate (Pierce, Rockford, IL, USA).

**Table 3:** Antibodies for Western blot

**Antibody**	**Host**	**Vendor**	**Dilution**	**Molecular weight (kDa)**
iNOS/NOS2	Rabbit	BD Biosciences	1:4000	130
α-Actinin	Mouse	BD Biosciences	1:5000	105
p- NFκB p65 (Ser536)	Rabbit	Cell Signaling	1:1000	65
NFκB p65	Rabbit	Cell Signaling	1:1000	65
p-PKR (Thr451)	Rabbit	Sigma-Aldrich	1:500	65
GAPDH	Mouse	Millipore	1:2000	36
p-eIF2α (Ser51)	Rabbit	Abcam	1:500	36
α-rabbit-HRP conjugated	Goat	Jackson ImmunoResearch	1:5000	
α-mouse-HRP conjugated	Goat	Jackson ImmunoResearch	1:2000	

Image densitometry was performed with a Bio-Rad GS-810 scanner (BIO-RAD Labs, Richmond, CA, USA) and analyzed with Quantity One 4.2 software (BIO-RAD). Glyceraldehyde-3-phosphate dehydrogenase (GAPDH) and α-actinin expression were used as loading control for microglia and astrocyte samples, respectively.

### Migration assays of microglial cells

Microglial cells were added on the upper part of a Transwell well (pore size 8-μm, Corning, San Dimas, CA, USA) in RPMI medium without FBS only or with TUDCA (200 μM or 100 μM) and IFN-γ (20 ng/mL) was used as chemoattractant and added to the lower well [27,28].

To study the influence of activated astrocytes on microglial cell migration, astrocytes were seeded on the wells. The next day, cells were preincubated with TUDCA (200 μM for 90 min) and then exposed to LPS (1 μg/mL) and IFN-γ (10 ng/mL) for 24 h. Supernatants were removed from the wells and astrocytes were washed twice with warm PBS. DMEM with 10% FBS was added to the astrocytes and incubated for an additional 24 h to obtain the conditioned media. After this period, Transwells were placed on the wells, microglial cells were seeded in the upper part of the Transwell and left for 24 h. Non-migrating cells were removed from the inserts with a cotton swab. Migrating microglial cells in the Transwells were fixed with 4% PFA for 15 min on ice, washed with PBS, and stained with Hoechst 33258 (1 μg/mL) for 5 min at room temperature. After washing with PBS, the number of attached cells in the lower part of the Transwells was determined by counting the Hoechst stained cells in photographs using an Olympus Provis AX70 microscope, coupled to an Olympus PD50 photography system. Each experiment was done in triplicate and photographs were obtained from five fields of each Transwell with a 20× microscope objective. Image J software was used to obtain the photographs and to analyse the images.

### Microglia proliferation assays

Microglial cells (20,000 cells/well) were seeded on 96-well plates and left overnight in an incubator at 37°C. The next day, the cells were pretreated for 2 h with concentrations of TUDCA ranging from 4 to 500 μM, and LPS (10 ng/mL) in RPMI medium supplemented with 5% FBS was added to the wells. After 48 h of treatment, proliferation was determined with the MTT assay (Sigma-Aldrich), according to the manufacturer’s protocol.

### Cytokine secretion assays

Astrocytes (500,000 cells/well) or microglial cells (2,000,000 cells/well) were seeded in 6-well plates and treated as previously described. After 6 or 24 h of treatment, supernatants were collected and processed according to the manufacturer’s instructions. Cytokines were measured using the commercial Quantibody® kit of Rat Cytokine Array 3 Glass Chip (Raybiotech Inc., Norcross, GA, USA).

### Statistical analysis

GraphPad Prism software version 5.0 for Windows was used for statistical analysis. The variances of the treatments were compared with a one-way ANOVA and the statistical significance between two experimental groups was determined by Mann-Whitney *U* test. Data in graphs are presented as the mean ± SD.

## Results

### TUDCA reduces microglial activation in the hippocampus of LPS-treated mice

**Figure 1:**
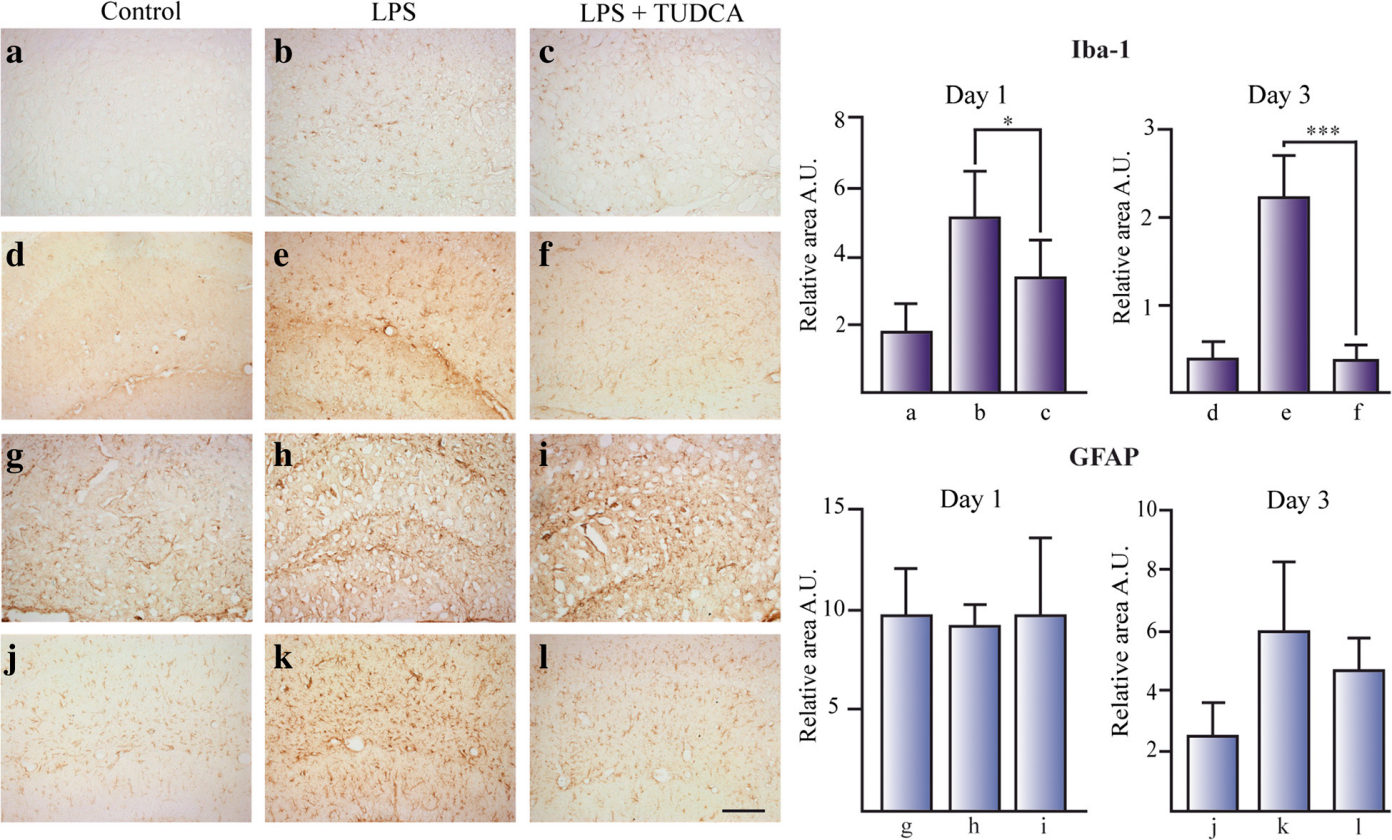
**TUDCA reduces microglial activation in the hippocampus of LPS treated mice.** The effect of TUDCA on glial activation was determined by the immunoreactive area for Iba-1 (for microglial cells) **(a–f)** and GFAP (for astrocytes) **(g–l)** related to total area in mice hippocampus icv injected with LPS. Section treatments are as follows: 1 day control **(a, g)**, 1 day icv LPS **(b, h)**, 1 day icv LPS + ip TUDCA **(c, i)**, 3 day control **(d, j)**, 3 day icv LPS **(e, k)**, and 3 day icv LPS + ip TUDCA **(f, l)**.*P <0.05, ***P <0.001. Scale bar represents 100 μm. The results represent the mean ± SD of at least 5 sections of 6 animals per group.

To study the effect of TUDCA on neuroinflammation, we used the inflammation model of unilateral icv injection of LPS in mice. GFAP (for astrocytes) and Iba-1 (for microglial cells) immunoreactivity were used to determine the glial reactivity in coronal sections from mice hippocampus. Iba-1 staining increased at 1 day (Figure 1a–c) and 3 days (Figure 1d–f) after LPS injection, compared to control animals. GFAP staining increased only at day 3 (Figure 1j–l). Mice with icv injection of LPS and treated with an ip injection of TUDCA slightly reduced Iba-1 immunoreactivity at day 1 (Figure 1b–c), compared to mice treated with LPS alone. Iba-1 immunoreactivity in mice with icv injection of LPS and treated with TUDCA reduced the immunoreactivity with respect to control animals at day 3 (Figure 1d–f). However, TUDCA did not have any effect on GFAP immunoreactivity (Figure 1g–l). In conclusion, TUDCA specifically reduced microglial reactivity in the hippocampus of LPS-treated mice.

### TUDCA reduces nitrite production in glial cell cultures by transcriptional inhibition of iNOS

**Figure 2:**
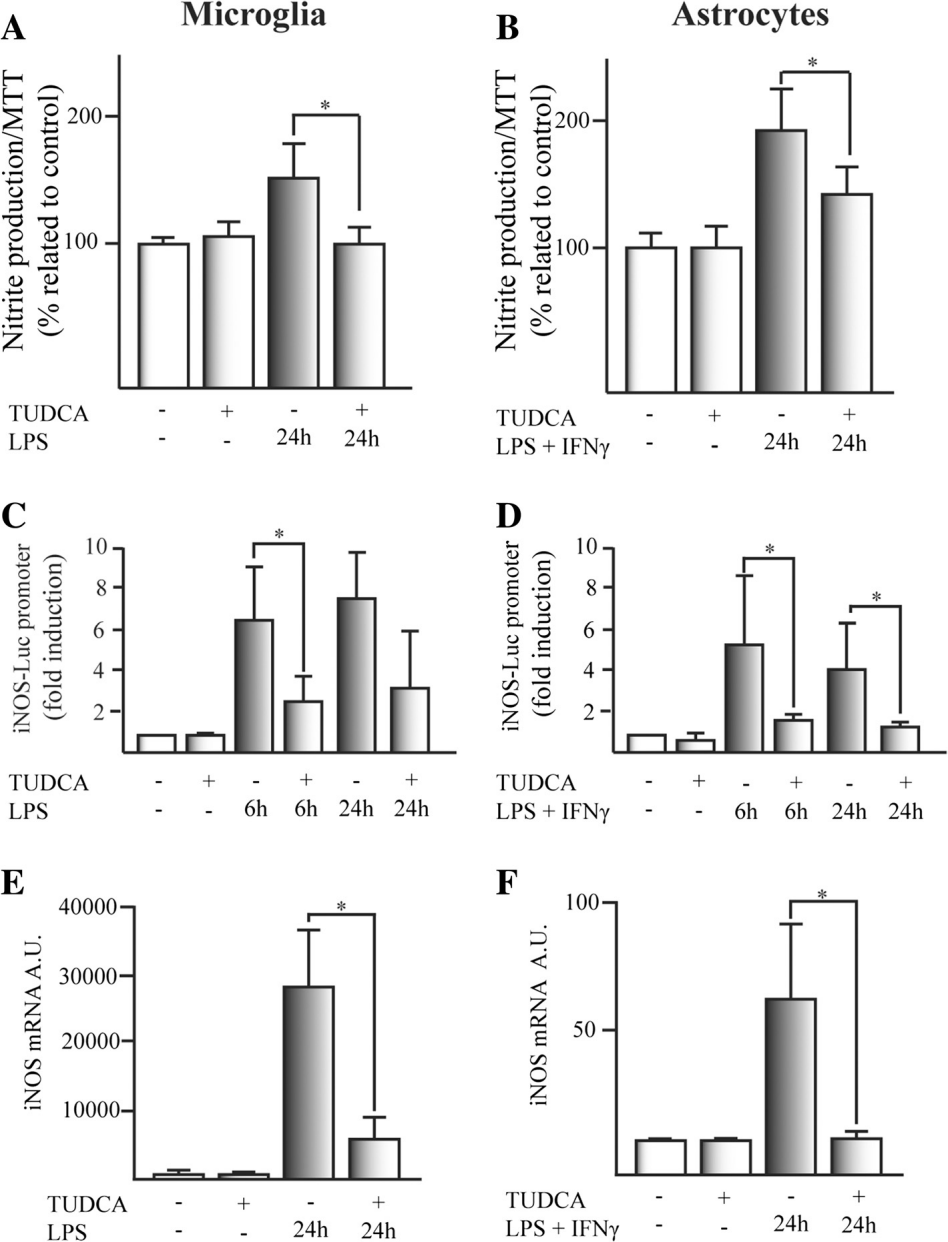
**TUDCA reduces proinflammatory stimuli-induced nitrite production in glial cell cultures through transcriptional inhibition of iNOS.** Nitrite production was determined in **(A)** microglial cells and **(B)** astrocytes. Cells were pretreated with TUDCA for 2 h and the proinflammatory stimuli was added and incubated for an additional 24 h. The results represent the mean of the percentage related to control ± SD of at least six experiments (microglial cells) and at least four experiments (in astrocytes) in triplicate. The effect of TUDCA on proinflammatory stimuli-induced luciferase activation of the rat iNOS-pGL3 firefly reporter was studied in **(C)** microglial cells and **(D)** astrocytes. SV40-pRL Renilla reporter was used as a control for transfection efficiency. The results represent the mean of the fold induction related to the control ± SD of at least four experiments in triplicate. The expression of the mRNA for iNOS was determined by qPCR in **(E)** microglial cells and **(F)** astrocytes. The expression of mRNA for β-actin and the expression of mRNA for 36B4 were used as loading control for astrocytes and microglial cells, respectively. The results represent the mean of the ratio between the expression of mRNA for iNOS/expression of mRNA for β-actin or 36B4 ± SD of at least three experiments in triplicate.*P <0.05, **P <0.01.

We studied nitrite production in glial cell cultures induced by proinflammatory stimuli to determine whether TUDCA has any effect on the inflammatory pathway. LPS-induced nitrite production in microglial cells was reduced to control levels by TUDCA (Figure 2A). In astrocytes, nitrite production induced by LPS plus IFN-γ was significantly reduced by TUDCA pretreatment (Figure 2B). Rat iNOS promoter-induced activity by LPS or LPS plus IFN-γ in glial cells, was reduced by TUDCA (Figure 2C and D). Moreover, iNOS mRNA transcription induced by LPS or LPS plus IFN-γ, was reduced in TUDCA treated cells, compared to control cells (Figure 2E and F). These results demonstrate that the inhibitory effect of TUDCA on nitrite production induced by LPS or LPS plus IFN-γ was mostly dependent on transcriptional regulation of iNOS.

### TUDCA inhibits proinflammatory stimuli-induced NFκB activation in glial cells

**Figure 3:**
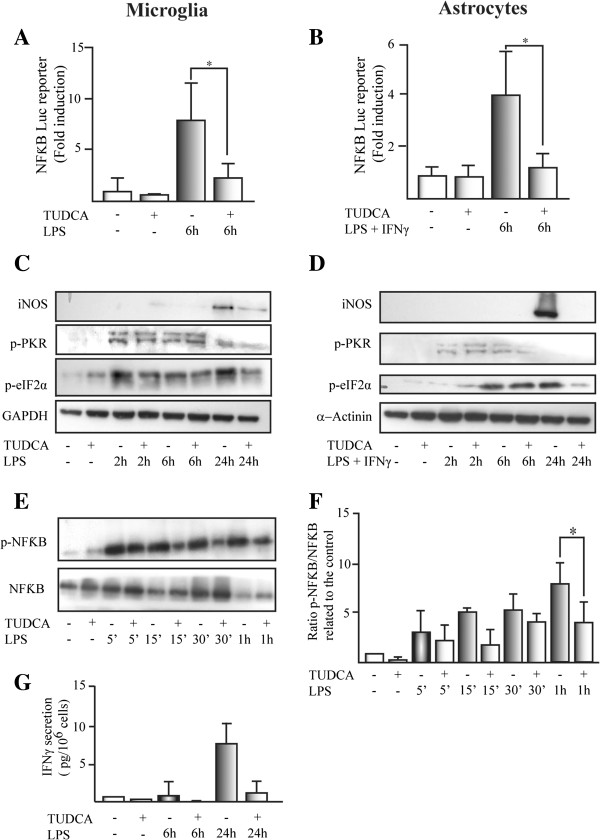
**TUDCA inhibits proinflammatory stimuli-induced NFκB activation in glial cells.** The effect of TUDCA on proinflammatory stimuli-induced luciferase activation of the NFκB-pGL3 firefly reporter was studied in **(A)** microglial cells and **(B)** astrocytes. SV40-pRL Renilla reporter was used as a control for transfection efficiency. The results represent the mean of the fold induction related to the control ± SD of at least four experiments in triplicate. The expression of iNOS and the phosphorylation status for PKR (p-PKR) and eIF2α (p-eIF2α) was studied by Western-blot in **(C)** microglial cells and **(D)** astrocytes. GAPDH and α-actinin were used as loading control for microglial cells and astrocytes, respectively. For band densitometries see Additional file 3. **(E)** NFκB activation was determined by the phosphorylation of p65 induced (p-NFκB) by LPS in microglial cells. The ratio between phosphorylated p65 related to the total p65 (NFκB) expression was presented in **(F)**. The results represent the mean of the fold induction related to the control for phosphorylated p65 (NFκB)/total p65 (NFκB) ± SD of four independent experiments. **(G)** The secretion of the cytokines IFN-γ (pg/10^6^ cells) was studied in the conditioned media of microglial cells. Data represent the mean (pg/10^6^ cells) ± SD of four experiments in triplicate.*P <0.05.

Activation of the NFκB proinflammatory pathway was studied in glial cells with transient transfection with an NFκB reporter plasmid [26]. Pretreatment with TUDCA reduced LPS (in microglial cells) and LPS plus IFN-γ (in astrocytes) induced NFκB reporter activation (Figure 3A and B). TUDCA had an effect on different proteins downstream of proinflammatory stimuli that activated pathways in glial cells (Figure 3C and D. For band densitometries check Additional file 3). Thus, iNOS protein expression and eukaryotic initiation factor 2 subunit alpha (eIF2α) phosphorylation at Serine 51, induced by proinflammatory stimuli, were reduced in TUDCA-pretreated microglial cells (Figure 3C) and astrocytes (Figure 3D). However, phosphorylation of protein kinase RNA-activated (PKR) at serine 451 induced by proinflammatory stimuli was not affected by TUDCA in both glial cell types (Figure 3C and D). LPS-induced NFκB p65 phosphorylation at serine 536 was reduced in microglial cells pretreated with TUDCA (Figure 3E and F). We studied the secretion of the proinflammatory cytokine IFN-γ, an NFκB regulated gene. LPS-induced IFN-γ secretion was completely inhibited by TUDCA pretreatment of microglial cells (Figure 3F). These results suggest that TUDCA affects proinflammatory pathways between PKR phosphorylation and NFκB phosphorylation.

### TUDCA reduces the activated microglia in the hippocampus of LPS treated mice

**Figure 4:**
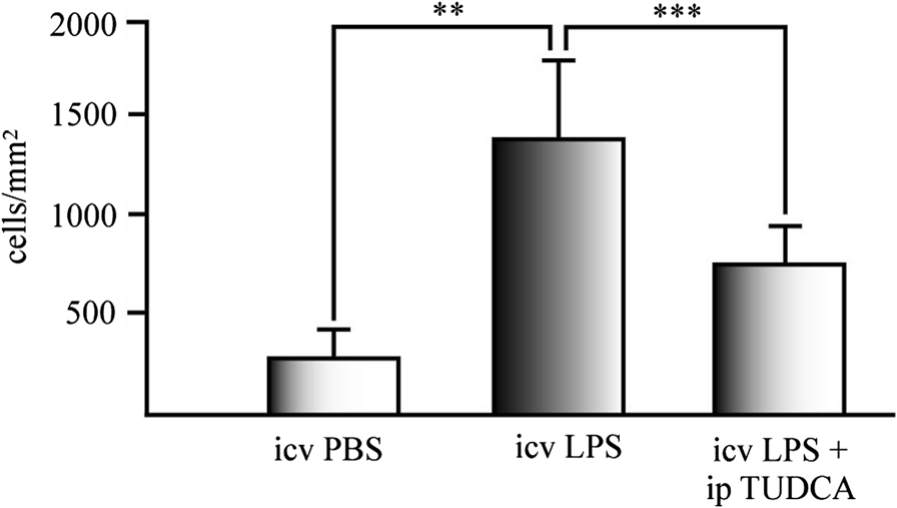
**TUDCA reduces activated microglia in the hippocampus of LPS treated mice.** Iba-1 positive cells were counted in hippocampal sections of treated mice after 3 days of icv injection of LPS. Data represents the mean of Iba-1 positive cells per mm^2^ of the mice hippocampus of five sections of at least six animals per experimental group. **P <0.01; ***P <0.001.

To determine whether the reduction of Iba-1 immunoreactivity in the hippocampus of TUDCA-treated mice compared to mice treated only with LPS (Figure 1a–f) could be due to reduction in Iba-1 positive microglial cells, we counted the number of positive cells per mm^2^ in all the experimental groups. As shown in Figure 4, mice pretreated with TUDCA and treated with icv injection of LPS considerably reduced the number of Iba-1 positive cells in the hippocampus, compared to the animal group treated with LPS alone. These data suggest that TUDCA reduced microglial activation in the hippocampus by reducing Iba-1 expression and/or reduces LPS-induced migration of hippocampal microglial cells or blood monocytes to neural parenchyma.

### TUDCA reduces microglia cell migration *in vitro*

**Figure 5:**
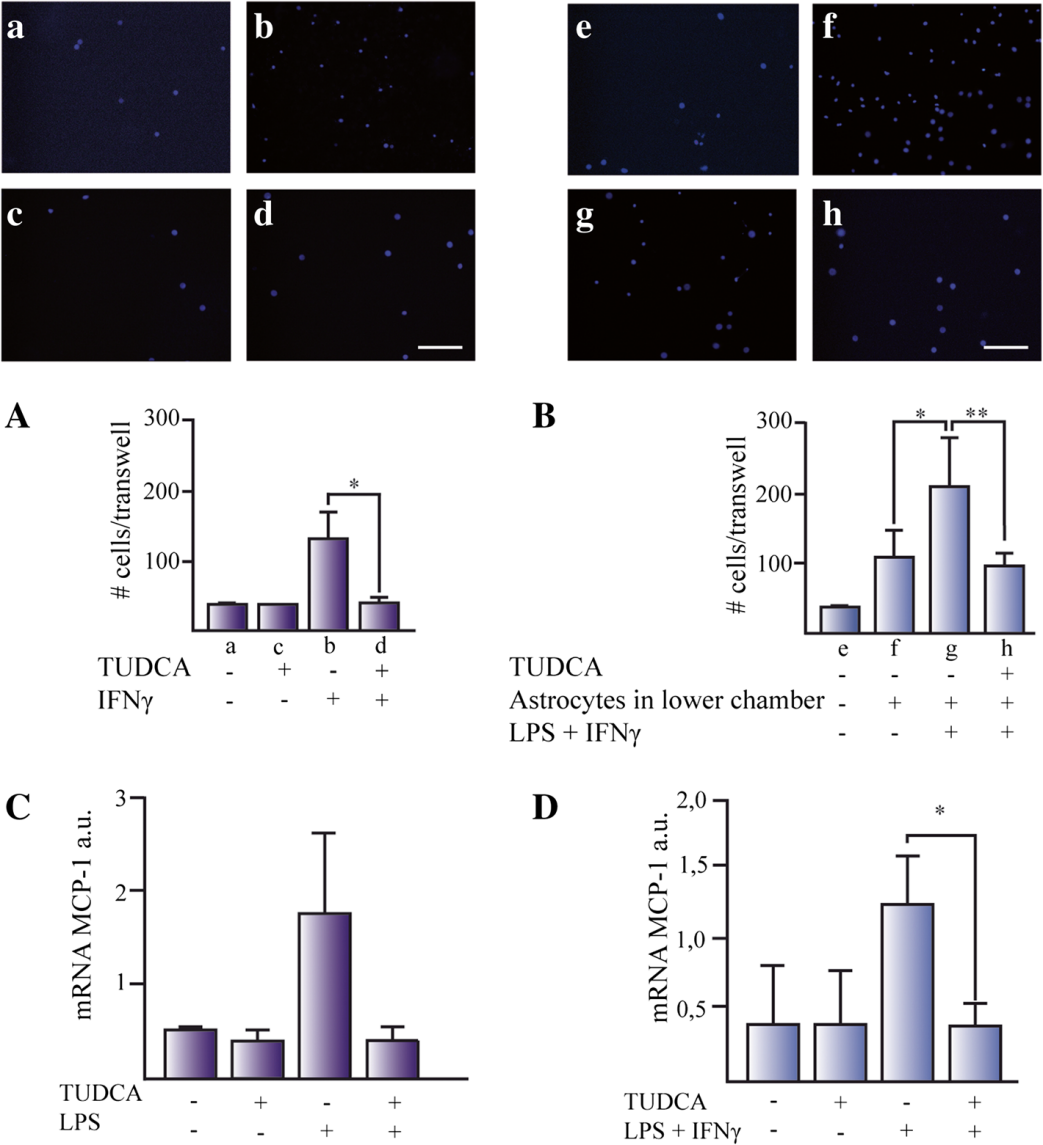
**TUDCA reduces microglia cell migration *in vitro*.** Microglial migration was studied using **(A)** IFN-γ and **(B)** conditioned media from proinflammatory stimuli-activated astrocytes after 24 h exposure. **(A)** IFN-γ was added in the lower part of the Transwell and microglial cells were seeded in the upper part. DAPI positive cells attached to the lower part of the Transwell were counted. Data represents the mean of the number of cells ± SD of at least five fields of three Transwells for each experimental group. Representative pictures were taken from cells treated with **(a)** control media, **(b)** control plus TUDCA, **(c)** IFN-γ, and **(d)** IFN-γ + TUDCA. **(B)** Microglial migration was studied in media conditioned for 24 h from: **(e)** control media without astrocytes, **(f)** untreated astrocytes, **(g)** astrocytes treated with LPS plus IFN-γ, and **(h)** astrocytes pretreated with TUDCA and treated with LPS plus IFN-γ. The expression of the mRNA for MCP-1 was determined by qPCR in **(C)** microglial cells and **(D)** astrocytes. The expression of mRNA for 36B4 and β-actin were used as loading control for microglial cells and astrocytes, respectively. The results represent the mean of the ratio between the expression of mRNA for iNOS/expression of mRNA for β-actin or 36B4 ± SD of at least three experiments in triplicates. *P <0.05. Scale bars represent 50 μm.

To test whether TUDCA had a direct effect on microglial migration, we used an *in vitro* Transwell assay and IFN-γ (20 ng/mL) as cell chemoattractant. We plated microglial cells on the upper chamber of the Transwell and IFN-γ was added to the lower chamber to build an appropriate gradient. There was a 4- to 6-fold increase in cell migration when treating cells with IFN-γ (Figure 5A). The effect of IFN-γ on microglial migration was completely abolished by TUDCA (Figure 5A).

Next, we examined the effect of activated astrocytes on microglial migration. Astrocytes were either just grown on DMEM and treated with LPS (1 μg/mL) plus IFN-γ (10 ng/mL) or pretreated with TUDCA and treated with LPS plus IFN-γ. Cells were washed twice with warm PBS to eliminate traces of the compounds and media were conditioned for 24 h. After this incubation period, microglia were seeded on the Transwells and left for 24 h. Astrocytes, *per se*, induced a 3-fold increase in microglial migration compared to controls without cells (Figure 5B). Astrocytes treated with LPS plus IFN-γ induced release of chemoattractant molecules to the media that led to an extra 2-fold increase of microglial migration rate compared to non-treated astrocyte conditioned media. Pre-treatment of astrocytes with TUDCA reverted microglial migration rates to the scores with non-activated astrocytes. These results suggest that TUDCA pretreatment reduced the expression of chemoattractants induced by the proinflammatory pathway. To test this possibility, we studied the transcriptional regulation of monocyte chemotactic protein-1 (MCP-1), one of the most relevant chemoattractant chemokines for microglial cells, by quantitative PCR in microglial cells (Figure 5C) and astrocytes (Figure 5D). In both cell types, proinflammatory pathway-induced transcriptional upregulation of MCP-1 was reduced by pretreating cells with TUDCA.

### TUDCA reduces VCAM-1 expression in the hippocampus of LPS treated mice

**Figure 6:**
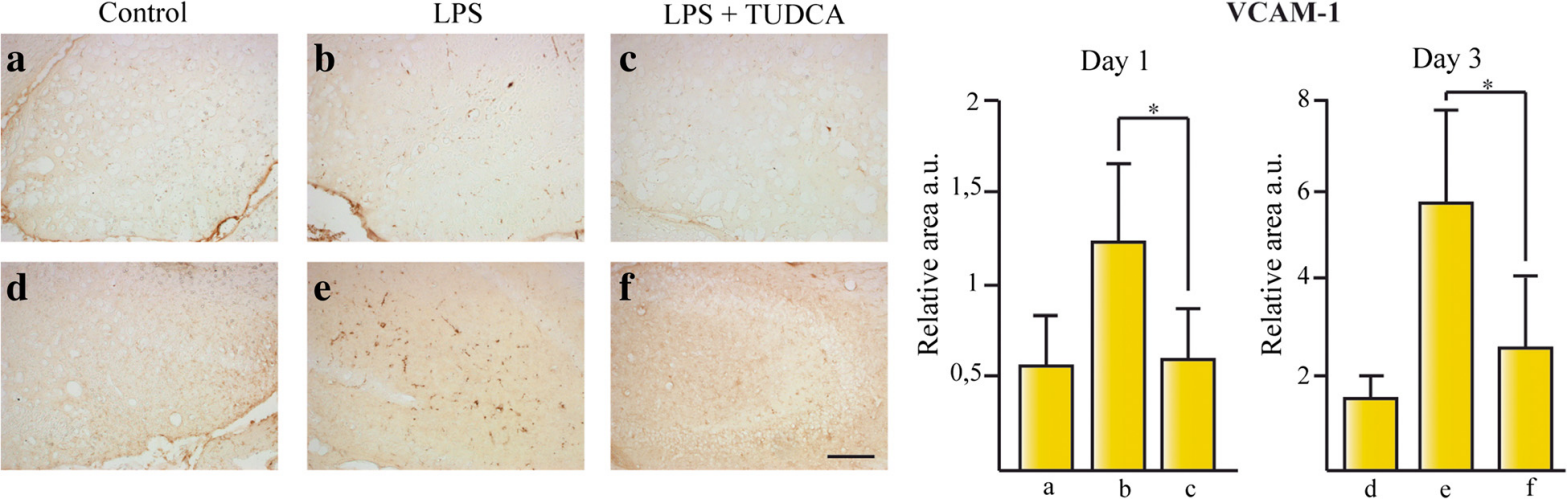
**TUDCA reduces VCAM-1 expression in the hippocampus of LPS treated mice.** The effect of TUDCA on vascular endothelium activation was determined by the immunoreactive area for VCAM-1 at 1 day **(a–c)** and 3 days **(d–f)** after icv injection with LPS related to total area in mice hippocampus. Section treatments are as follows: **(a)** untreated animals control for 1 day treatment, **(b)** animals sacrificed 1 day after icv injection of LPS, **(c)** animals sacrificed 1 day after icv injection of LPS and treated with ip injections of TUDCA, **(d)** untreated animals sacrificed after 3 days, **(e)** animals sacrificed 3 days after icv injection of LPS, and **(f)** animals sacrificed 3 days after icv injection of LPS and treated with ip injections of TUDCA.*P <0.05, ***P <0.001. Scale bar represents 100 μm. The results represent the mean ± SD of at least five sections of six animals per group.

As Iba-1 positive microglial cells were reduced in animals treated with TUDCA and icv injected with LPS, compared to the animal group treated with LPS alone (Figure 4), we tested whether the immunoreactivity of the Vascular Cell Adhesion 1 (VCAM-1), one of the most important adhesion proteins required for blood monocyte transmigration across the BBB, was affected in these mice (Figure 6). VCAM-1 was induced significantly after icv LPS injection, as early as 24 h, and there was a 5-fold VCAM-1 increase compared to control animals after 3 days. This increase was reverted in both time points when TUDCA was administered to the treated mice (Figure 6a–f). In conclusion, TUDCA reduced the activation of the proinflammatory pathway and probably reduced blood monocyte transmigration to neural parenchyma.

Our results support TUDCA might be a beneficial therapy to control neuroinflammatory process in neurological disorders.

## Discussion

Although the neuroprotective effects of bile acids have already been previously described, little is known about the effect of TUDCA on the neuroinflammatory pathway in glial cells. We have demonstrated that microglial activation is reduced by TUDCA in an animal model of acute neuroinflammation by LPS icv injection (Figure 1a–f). However, we do not see this effect in astrocytes at day 1 and day 3 after icv of LPS (using GFAP as a marker for activated astrocytes, Figure 1g–l). We cannot be sure that there is no effect of TUDCA on astrocyte activation because we have not tested other time points after 3 days of LPS injection.

We tested whether there was a direct effect on glial cell activation of proinflammatory stimuli, on astrocytes and microglial cell cultures. Our *in vitro* results showed that TUDCA pretreated cells decreased proinflammatory stimuli-induced nitrite production in astrocytes and microglial cells (Figure 2A and B). The production of nitric oxide in glial cells is mediated by the iNOS enzyme. We have demonstrated that the expression of iNOS enzyme is reduced at transcriptional (Figure 2C–F) and translational level (Figure 3C and D) by this bile conjugate after inflammatory stimulation in glial cells. The induction of iNOS expression is regulated by NFκB proinflammatory pathway [25]. We have shown that the expression (e.g., MCP-1 and VCAM-1) and secretion (e.g., IFN-γ) of other NFκB target genes are reduced by TUDCA. The activation of NFκB pathway by proinflammatory stimuli is affected on TUDCA pretreated cells, reducing NFκB phosphorylation (Figure 3E and F) and the activation of the NFκB reporter (Figure 3A and B).

How is TUDCA affecting the NFκB pathway? Our results indicate that it is not affecting PKR phosphorylation in threonine 451 induced by proinflammatory stimuli. However, TUDCA reduces the phosphorylation in Serine 51 in the translation initiator factor eIF2α (Figure 3C and D), as well as in NFκB p65 phosphorylation. The processes affected by TUDCA in the proinflammatory pathway are downstream PKR phosphorylation and upstream of NFκB p65 and eIF2α phosphorylation. In previous work, Joo et al. [19] showed that the microglial cell line BV-2, pretreated with a structurally similar bile salt to TUDCA, called UDCA, reduced nitrite production induced by β-amyloid. This effect was mediated through inhibition of IκB degradation that blocked NFκB activation. LPS-induced activation of NFκB is PKR dependent in alveolar macrophages [29]. PKR can stimulate NFκB activity by interacting with the IkappaB kinase complex, independently of PKR kinase activity and PKR-induced phosphorylation of eIF2α through IKKβ interaction [30]. TUDCA might inhibit PKR-IKKβ interaction or a process downstream that reduces NFκB phosphorylation. As TUDCA reduces eIF2α phosphorylation too, it might be a common process related to the phosphorylation of both proteins. As the translational repression induced by eIF2α phosphorylation is required for NFκB activation [31], TUDCA might be targeting the phosphorylated status of eIF2α, activating serine phosphatases, and reducing phosphorylated eIF2α and NFκB.

The reduction of Iba-1 positive cells in the hippocampus of mice treated with TUDCA plus LPS (Figure 4) suggested that this effect could be due to a reduction in the microglial migratory capacity. To test this possibility, we studied the effect of TUDCA on IFN-γ-induced migration in microglial cell cultures (Figure 5A). TUDCA inhibited IFN-γ-induced migration of microglial cells to control levels. Moreover, TUDCA inhibited the migration of microglial cells induced by astrocytes activated by proinflammatory stimuli (Figure 5B). As the chemokine MCP-1 is an important regulator of microglial migration, we tested MCP-1 expression in glial cells. TUDCA reduced MCP-1 transcription induced by proinflammatory stimuli in microglial cells (Figure 5C) and astrocytes (Figure 5D).

As the expression of the VCAM-1 protein in the CNS endothelium is critically involved in blood monocyte transmigration into the neural parenchyma [32], we studied the immunoreactivity for this protein in our animal model of acute neuroinflammation. TUDCA reduced LPS-induced VCAM-1 expression at day 1 and day 3 after LPS injection, compared to animals treated with LPS alone (Figure 6). This result suggests that TUDCA reduced endothelium activation by LPS and, as a consequence, might reduce blood monocyte migration into the CNS parenchyma.

Our results demonstrate that TUDCA reduced glial cell activation induced by proinflammatory stimuli at least by inhibiting NFκB activation. As TUDCA reduced NFκB activation induced by proinflammatory stimuli, it inhibited different key proteins involved in other NFκB regulated processes, such as microglial migration (e.g., MCP-1) and endothelium activation (e.g., VCAM-1), by proinflammatory stimuli required for blood leukocyte transmigration to the CNS parenchyma.

## Conclusions

TUDCA is a neuroprotective agent in different animal models of stroke and neurological diseases. Nevertheless, little is known about the anti-inflammatory properties of TUDCA in the CNS. Our results suggest that TUDCA reduced glial cell activation induced by proinflammatory stimuli through inhibition of NFκB activity. TUDCA has a triple inhibitory effect on glial cells in the CNS parenchyma, inhibiting NFκB by i) reducing glial cell activation, ii) reducing microglial cell migratory capacity, and iii) reducing the expression of chemoattractants (e.g., MCP-1) and vascular adhesion proteins (e.g., VCAM-1) required for microglial migration and blood monocyte invasion of the CNS inflammation site. Our results suggest a novel TUDCA anti-inflammatory mechanism with therapeutic implications for inflammatory diseases of the CNS.

## Abbreviations

BBB: Blood-brain barrier; CNS: Central nervous system; DMEM: Dulbecco’s modified Eagle’s medium; eIF2α: Eukaryotic initiation factor 2 subunit alpha; FBS: Foetal bovine serum; GFAP: Glial fibrillary acidic protein; Iba-1: Ionized calcium-binding adapter molecule 1; icv: Intracerebroventricular; IFN-γ: Interferon gamma; iNOS: Inducible nitric oxide synthase; ip: Intraperitoneal; LPS: Lipopolysaccharide; MCP-1: Monocyte chemotactic protein-1; MTT: 3-(4,5-dimethylthiazol-2-yl)-2,5-diphenyltetrazolium bromide; PBS: Phosphate-buffered saline; PFA: Paraformaldehyde; PKR: Protein kinase RNA-activated; P/S: Penicillin/streptomycin; qPCR: Quantitative polymerase chain reaction; RPMI: Roswell Park Memorial Institute medium 1640; SD: Standard deviation; SEM: Standard error of mean; TUDCA: Tauroursodeoxycholic acid; UDCA: Ursodeoxycholic acid; VCAM-1: Vascular cell adhesion molecule 1.

## Competing interests

The authors declare that they have no competing interests.

## Authors’ contributions

NYC performed the experiments, participated in the interpretation of the data, performed the statistical analysis, and drafted the manuscript. MABM participated in some experiments and in the revision of the manuscript draft. MNS participated in the design of the study, interpretation of the data, and in the revision of the manuscript draft. LRR conceived and designed the study, participated in the interpretation of the data, performed some experiments, and drafted the manuscript. All authors read and approved the final manuscript.

## Supplementary Material

Additional file 1 **Non-specific binding of the anti-mouse secondary antibody in mice hippocampus.** The secondary anti-mouse biotinylated antibody does not have any non-specific staining in mice hippocampus in acute inflammatory injury. Section treatments are as follows: Control **(a)**, icv LPS **(b)**, and icv LPS + ip TUDCA **(c)**. Scale bar 100 μm. Click here for file

Additional file 2 **Development of an *in vitro *proinflammatory response in microglia and astrocytes.** Cell treatment with increasing concentrations of LPS or LPS plus IFN-γ for 24 h showed that LPS alone induced nitrite secretion in microglial cells; however, astrocytes also required the addition of IFN-γ (20 ng/mL) to develop this response *in vitro*. Based on these results, we decided that the optimal LPS concentration to treat microglial cells was 200 ng/mL, because it induced an almost two-fold increase in the secretion of nitric oxide. We also decided to treat astrocytes with 1 μg/mL LPS + 20 ng/mL IFN-γ for the same reason. The bar graphs represent the mean of the percentage related to control ± SD of four experiments (for both cell types) in triplicate. Click here for file

Additional file 3 **TUDCA regulates the activation of NFκB proinflammatory pathway.** We studied the expression of iNOS and the phosphorylation level of eIF2α and PKR. In microglial cells, TUDCA downregulates the phosphorylation of eIF2α after 2 h of induction with LPS **(A)**, it does not affect the phosphorylation of PKR **(C)**, and reduces the iNOS expression at 24 h **(E)**. In astrocytes, TUDCA reduces the phosphorylation of eIF2α at 24 h after LPS treatment **(B)**, PKR phosphorylation is not affected by TUDCA **(D)**, and iNOS expression is also reduced at the same time point as microglia. The bar graphs represent the mean of the densitometry of the bands ± SEM of the phosphorylated form (for eIF2α and PKR) or iNOS protein expression normalized to the loading control (GAPDH for microglia and α-actinin for astrocytes) for three independent experiments. Click here for file
