# Supplementary material for: Tauroursodeoxycholic acid reduces glial cell activation in an animal model of acute neuroinflammation
Source: J Neuroinflammation. 2014 Mar 19;11:50. doi: 10.1186/1742-2094-11-50 (PMC4000131; doi:10.1186/1742-2094-11-50)
Supplement: Additional file 1 — Non-specific binding of the anti-mouse secondary antibody in mice hippocampus. The secondary anti-mouse biotinylated antibody does not have any non-specific staining in mice hippocampus in acute inflammatory injury. Section treatments are as follows: Control (a), icv LPS (b), and icv LPS + ip TUDCA (c). Scale bar 100 μm. [file 1742-2094-11-50-S1.pdf]

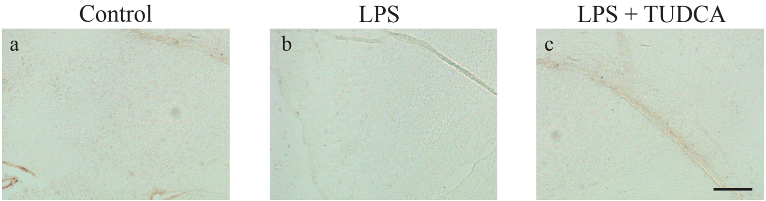

Additional file A2. Nonspecific binding of the anti-mouse secondary antibody in mice hippocampus. The secondary anti-mouse biotinylated antibody does not have any nonspecific staining in mice hippocampus in acute inflammatory injury. Section treatments are as follows: Control (a) , icv LPS (b), icv LPS + ip TUDCA (c). Scale bar 100 μm.
