# Supplementary material for: Tauroursodeoxycholic acid reduces glial cell activation in an animal model of acute neuroinflammation
Source: J Neuroinflammation. 2014 Mar 19;11:50. doi: 10.1186/1742-2094-11-50 (PMC4000131; doi:10.1186/1742-2094-11-50)
Supplement: Additional file 2 — Development of an in vitro proinflammatory response in microglia and astrocytes. Cell treatment with increasing concentrations of LPS or LPS plus IFN-γ for 24 h showed that LPS alone induced nitrite secretion in microglial cells; however, astrocytes also required the addition of IFN-γ (20 ng/mL) to develop this response in vitro. Based on these results, we decided that the optimal LPS concentration to treat microglial cells was 200 ng/mL, because it induced an almost two-fold increase in the secretion of nitric oxide. We also decided to treat astrocytes with 1 μg/mL LPS + 20 ng/mL IFN-γ for the same reason. The bar graphs represent the mean of the percentage related to control ± SD of four experiments (for both cell types) in triplicate. [file 1742-2094-11-50-S2.pdf]

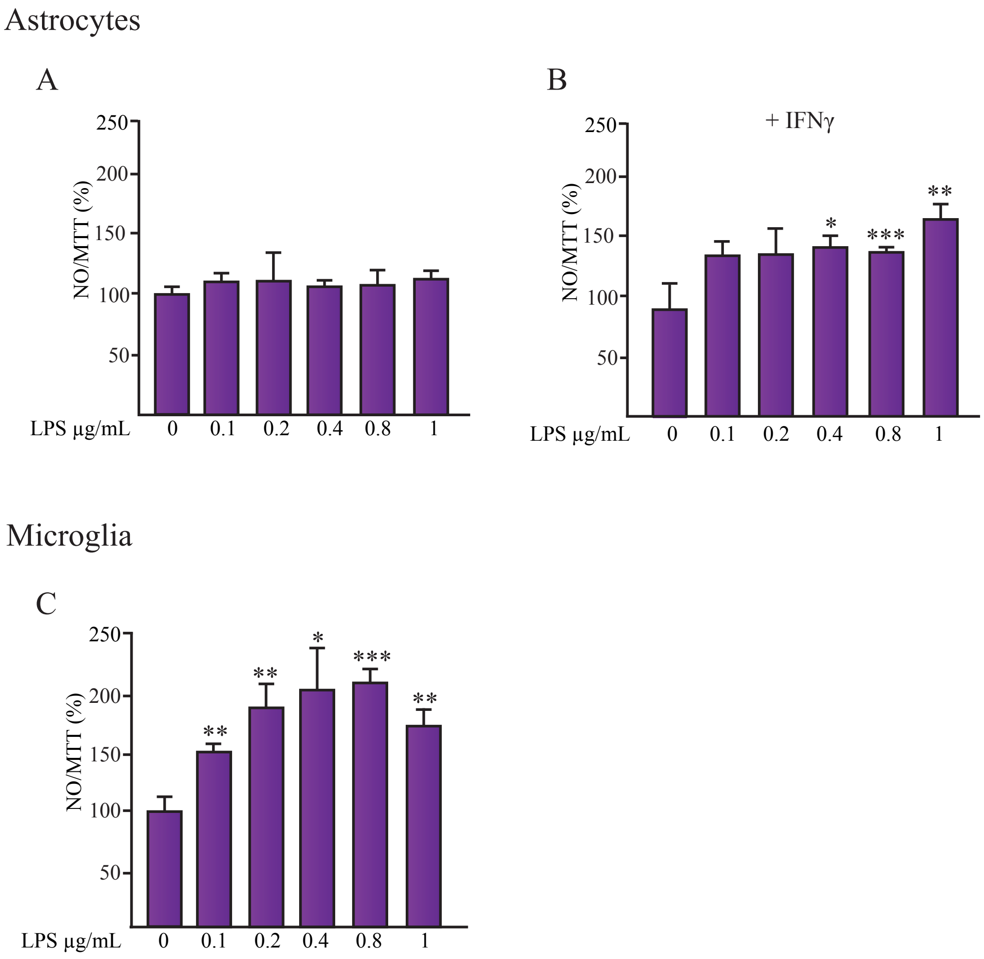

Additional file A1. Development of an in vitro proinflammatory response in microglia and astrocytes. Cell treatment with increasing concentrations of LPS or LPS plus IFNγ for 24h showed that LPS alone induced nitrite (NO) secretion in microglial cells; however, astrocytes also required the addition of IFNγ (20ng/mL) to develop this response in vitro. Based on these results, we decided that the optimal LPS concentration to treat microglial cells was 200ng/mL, because it induced an almost two fold increase in the secretion of NO. We also decided to treat astrocytes with 1µg/mL LPS + 20ng/mL IFNγ, for the same reason. We discarded lower concentrations that were also inducing NO secretion in order to guarantee a proper proinflammatory response and be able to study the effect of a bile acid (TUDCA) in this response. The bar graphs represent the mean of the percentage related to control ± SD of 4 experiments (for both cell types) in triplicates.
