# Supplementary material for: Tauroursodeoxycholic acid reduces glial cell activation in an animal model of acute neuroinflammation
Source: J Neuroinflammation. 2014 Mar 19;11:50. doi: 10.1186/1742-2094-11-50 (PMC4000131; doi:10.1186/1742-2094-11-50)
Supplement: Additional file 3 — TUDCA regulates the activation of NFκB proinflammatory pathway. We studied the expression of iNOS and the phosphorylation level of eIF2α and PKR. In microglial cells, TUDCA downregulates the phosphorylation of eIF2α after 2 h of induction with LPS (A), it does not affect the phosphorylation of PKR (C), and reduces the iNOS expression at 24 h (E). In astrocytes, TUDCA reduces the phosphorylation of eIF2α at 24 h after LPS treatment (B), PKR phosphorylation is not affected by TUDCA (D), and iNOS expression is also reduced at the same time point as microglia. The bar graphs represent the mean of the densitometry of the bands ± SEM of the phosphorylated form (for eIF2α and PKR) or iNOS protein expression normalized to the loading control (GAPDH for microglia and α-actinin for astrocytes) for three independent experiments. [file 1742-2094-11-50-S3.pdf]

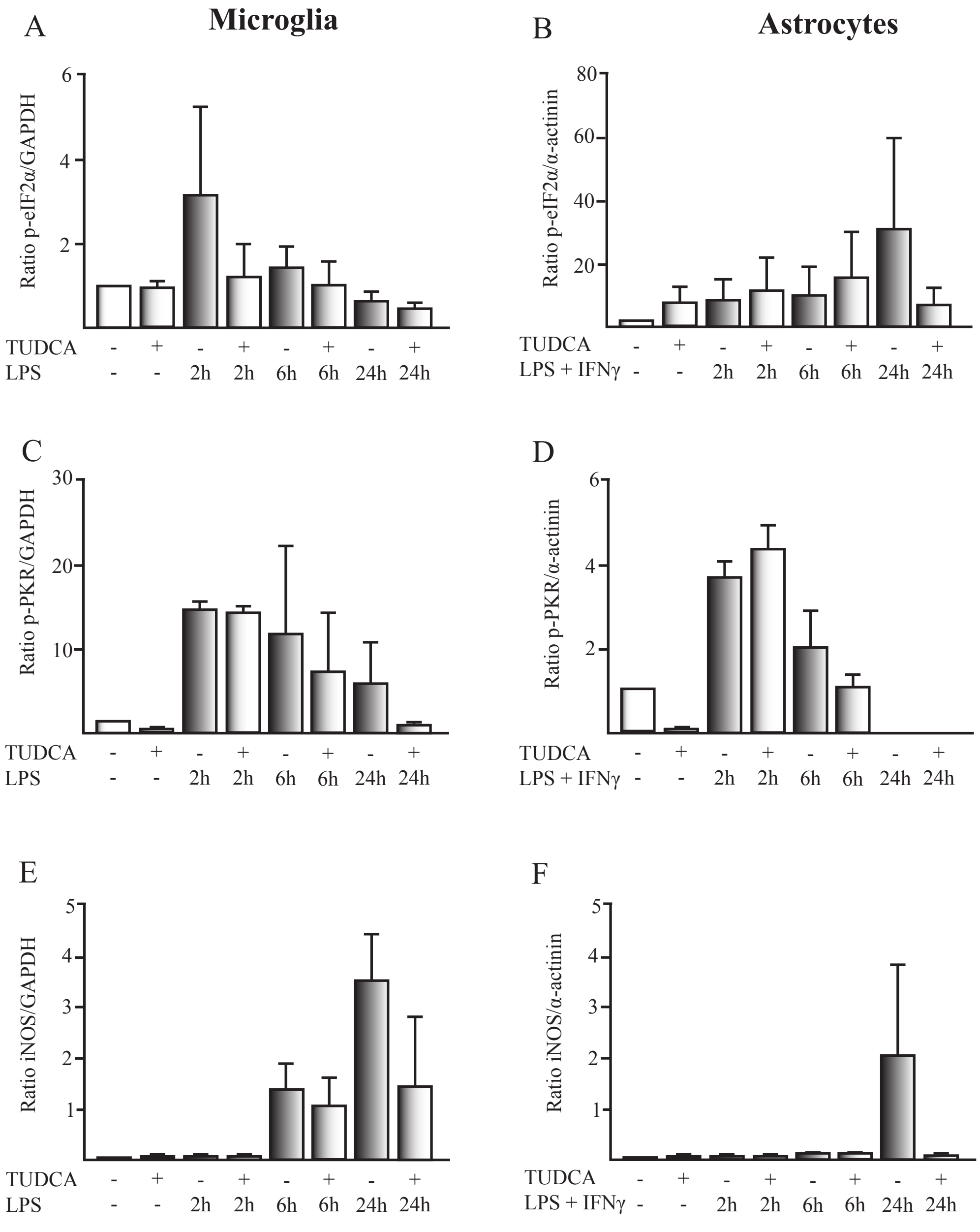

Additonal file A3: TUDCA regulates the activation of NFκB proinflammatory pathway. We studied the expression of iNOS and the phosphorylation level of eIF2α and PKR. In microglial cells, TUDCA downregulates the phosphorylation of eIF2α after 2h of induction with LPS (A), it does not affect the phosphorylation of PKR (C), and reduces the iNOS expression at 24h (E). In astrocytes, TUDCA reduces the phosphorylation of eIF2α at 24h after LPS treatment (B), PKR phosphorylation is not affected by TUDCA (D), and iNOS expression is also reduced at the same time point as microglia. The bar graphs represent the mean of the densitometry of the bands ± SEM of the phosphorylated form (for eIF2α and PKR) or iNOS protein expression normalized to the loading control (GAPDH for microglia and α-actinin for astrocytes) for 3 independent experiments.
